# Entorhinal Denervation Induces Homeostatic Synaptic Scaling of Excitatory Postsynapses of Dentate Granule Cells in Mouse Organotypic Slice Cultures

**DOI:** 10.1371/journal.pone.0032883

**Published:** 2012-03-05

**Authors:** Andreas Vlachos, Denise Becker, Peter Jedlicka, Raphael Winkels, Jochen Roeper, Thomas Deller

**Affiliations:** 1 Institute of Clinical Neuroanatomy, Neuroscience Center, Goethe-University Frankfurt, Frankfurt, Germany; 2 Institute of Neurophysiology, Neuroscience Center, Goethe-University Frankfurt, Frankfurt, Germany; McGill University, Canada

## Abstract

Denervation-induced changes in excitatory synaptic strength were studied following entorhinal deafferentation of hippocampal granule cells in mature (≥3 weeks old) mouse organotypic entorhino-hippocampal slice cultures. Whole-cell patch-clamp recordings revealed an increase in excitatory synaptic strength in response to denervation during the first week after denervation. By the end of the second week synaptic strength had returned to baseline. Because these adaptations occurred in response to the loss of excitatory afferents, they appeared to be in line with a homeostatic adjustment of excitatory synaptic strength. To test whether denervation-induced changes in synaptic strength exploit similar mechanisms as homeostatic synaptic scaling following pharmacological activity blockade, we treated denervated cultures at 2 days post lesion for 2 days with tetrodotoxin. In these cultures, the effects of denervation and activity blockade were not additive, suggesting that similar mechanisms are involved. Finally, we investigated whether entorhinal denervation, which removes afferents from the distal dendrites of granule cells while leaving the associational afferents to the proximal dendrites of granule cells intact, results in a global or a local up-scaling of granule cell synapses. By using computational modeling and local electrical stimulations in Strontium (Sr^2+^)-containing bath solution, we found evidence for a lamina-specific increase in excitatory synaptic strength in the denervated outer molecular layer at 3–4 days post lesion. Taken together, our data show that entorhinal denervation results in homeostatic functional changes of excitatory postsynapses of denervated dentate granule cells in vitro.

## Introduction

Denervation-induced plasticity is a form of neuronal plasticity that is of particular interest in the context of neurological diseases. Since neurons are highly interconnected cells, the degeneration of a given neuronal population will inevitably result in the denervation of its target neurons. If this denervation is sufficiently extensive, transneuronal changes of the denervated neurons may occur, ranging from spine loss and dendritic atrophy to cell death [1], [2]. Thus, secondary neuronal damage may follow neuronal degeneration and this secondary damage may contribute to the clinical symptoms of the disease as well as disease progression [3]. The loss of afferents, however, also induces other plastic changes such as collateral sprouting of the remaining axons and reactive synaptogenesis [1], [2]. These denervation-induced forms of neuronal plasticity compensate at least in part for the loss of afferent innervation and may play a pivotal role for the functional recovery of denervated neurons following denervation.

In recent years a new plasticity mechanism has been identified, which compensates for changes in afferent neuronal activity by homeostatically scaling the strength of synapses to keep the afferent drive of a neuron within a physiological range [4], [5]. A reduction in afferent drive, which can be achieved by treatment with the sodium channel blocker tetrodotoxin (TTX), will thus result in a strengthening of excitatory synapses [6]. Since axonal denervation results in the loss of synapses, we hypothesized that this plasticity mechanism, i.e., homeostatic synaptic scaling, could also play a role following deafferentation. To assess the effects of partial deafferentation on excitatory synaptic strength, we employed the versatile in vitro entorhinal lesion model [7], which exhibits many of the features seen in vivo after entorhinal denervation, including axonal sprouting [8] and dendritic reorganization [9], [10]. In this model entorhinal denervation results in a layer-specific loss of synapses in the outer parts of the molecular layer of the dentate gyrus while leaving afferent synapses to the inner parts of the molecular layer intact [1], [2], [11]. Accordingly, the question can be addressed whether entorhinal denervation elicits changes in synaptic strength of denervated granule cells and whether these changes affect all synapses or only those located in the outer parts of the molecular layer.

Whole-cell patch-clamp recordings of denervated and non-denervated granule cells disclosed a denervation-induced increase in excitatory synaptic strength. By combining entorhinal deafferentation with TTX-treatment we acquired experimental evidence that denervation induces homeostatic up-scaling of excitatory granule cell postsynapses. At 3–4 days post lesion (dpl) the increase in synaptic strength was restricted to synapses located on denervated dendritic segments in the outer parts of the molecular layer, demonstrating that deafferentation results in a local strengthening of surviving granule cell synapses. Taken together, our results suggest that homeostatic plasticity mechanisms, such as homeostatic synaptic scaling, can partially compensate for the denervation-induced loss in afferent drive and can thus be expected to play a role in the response of neurons to denervation-induced damage.

## Results

### Entorhinal cortex lesion in vitro

**Figure 1:**
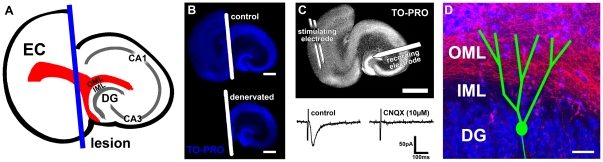
Entorhinal denervation in vitro leads to a layer-specific loss of excitatory input. (**A**) Schematic of an organotypic entorhino-hippocampal slice culture. The entorhino-hippocampal fiber tract (red) terminating in the outer molecular layer (OML) of the dentate gyrus (DG) and the plane of transection (blue) are illustrated. (EC, entorhinal cortex; IML, inner molecular layer). (**B**) Control and denervated cultures (blue, TO-PRO nuclear stain; white, plane of transection). In all denervation experiments the EC was removed. This procedure does not damage the DG. Scale bar: 500 µm. (**C**) Electrical stimulations of the entorhinal cortex while recording evoked EPSCs from dentate granule cells revealed an intact and functional entorhino-hippocampal projection. The evoked EPSCs were blocked by application of the AMPA-receptor antagonist CNQX (10 µM). Scale bar: 500 µm. (**D**) Entorhinal denervation leads to a layer-specific loss of excitatory input in the OML, while dendritic segments in the IML are not denervated (red, Mini-Rubi traced entorhinal fibers; green, schematic of a dentate granule cell). Scale bar: 50 µm.

Three weeks old entorhino-hippocampal slice cultures (18–20 days in vitro; div) were used in the experiments. The entorhinal cortex was cut away from the hippocampus and removed from the culture dish using a sterile scalpel (Figure 1A, B). This lesion does not damage the granule cells or their dendrites in the dentate gyrus [9], but results in a layer-specific loss of excitatory entorhinal afferents to the outer molecular layer (OML) of the dentate gyrus (Figure 1C, D; see also [9], [10]). Associational fibers which arise from glutamatergic mossy cells in the hilus and which terminate in the inner molecular layer (IML) of the dentate gyrus are not injured by the lesion [8], [9], [12], [13].

### Denervation induces an increase in excitatory synaptic strength

**Figure 2:**
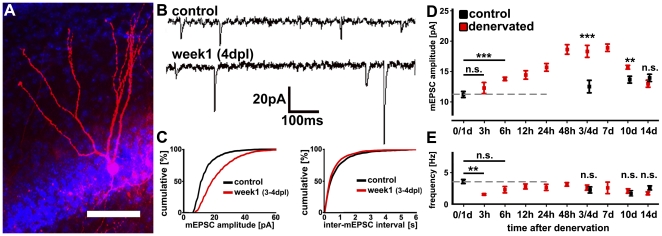
Denervation induces an increase in excitatory synaptic strength. (**A**) Granule cell in a denervated culture at 3–4 days post lesion (dpl). The neuron was filled with biocytin and post-hoc identified using Alexa568-conjugated streptavidin (red; blue, TO-PRO nuclear stain). Scale bar: 50 µm. (**B**) Sample traces of miniature excitatory post synaptic currents (mEPSC) recordings from non-lesioned control cultures and from denervated granule cells at 4 dpl. (**C**) Cumulative distribution diagrams of mEPSC amplitudes and inter-event intervals from control and denervated (3–4 dpl) dentate granule cells revealed a significant increase in amplitude after denervation. (**D, E**) mEPSCs were recorded at 3 h, 6 h, 12 h, 24 h, 48 h, 3–4 dpl, 7 dpl, 10 dpl and 14 dpl. Age-matched controls were recorded at 0–1 d, 3–4 d, 10 d and 14 d (n = 4–11 cultures per group; ≥3 neurons per culture; 165 neurons total). These experiments demonstrated increased mEPSC amplitudes between 6 h and 10 dpl (D). The frequency of mEPSC events was initially decreased (at 3 h post lesion) and subsequently recovered back to control levels (E).

To assess the effects of entorhinal denervation on glutamatergic synaptic strength, miniature excitatory postsynaptic currents (mEPSCs) were recorded from control and denervated granule cells using whole-cell patch-clamp recordings at 3 h, 6 h, 12 h, 24 h, 48 h, 3–4 dpl, 7 dpl, 10 dpl and 14 dpl (age-matched controls were recorded at 0–1 d, 3–4 d, 10 d and 14 d; n = 4–11 cultures per group; ≥3 neurons recorded per culture; 165 neurons total; Figure 2). Recorded neurons were filled with biocytin, post hoc stained and identified using morphological criteria (Figure 2A). A significant increase in the mean mEPSC amplitude of denervated granule cells compared to age-matched non-denervated controls was seen between 6 h and 10 dpl (Figure 2D). At 14 dpl no significant difference between denervated and non-denervated granule cells was observed (control 14 d: 13,9±0.60 pA; denervated 14 dpl: 13,07±0.59 pA; p = 0.33). The frequency of mEPSC events was initially reduced (at 3 h post lesion; control: 3.5±0.3 Hz; denervated: 1.6±0.1 Hz; p<0.01) and recovered to control levels (Figure 2E). We concluded that partial denervation of dentate granule cells in vitro induces an increase in excitatory synaptic strength, which returns to baseline between 10 and 14 dpl.

Because the adaptation of synaptic strength occurred in response to the loss of excitatory entorhinal afferents, it appeared to be in line with a compensatory adjustment in excitatory synaptic strength, i.e., homeostatic synaptic up-scaling, which is observed in other models after a prolonged reduction in the cell's afferent activity [4], [5], [14]–[16].

### Denervation induces a compensatory increase in excitatory synaptic strength similar to TTX

**Figure 3:**
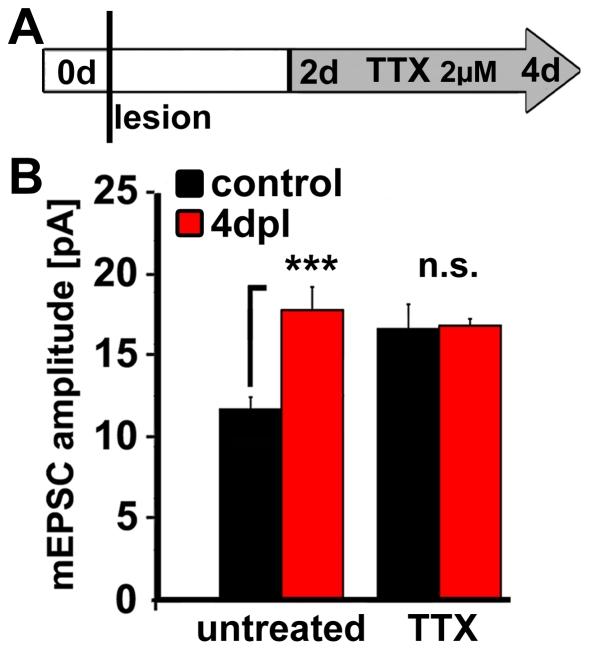
Denervation induces homeostatic synaptic scaling. (**A, B**) Treatment of denervated cultures with the sodium channel blocker tetrodotoxin (A; TTX 2 µM, 2 d) did not significantly change the denervation-induced compensatory increase in excitatory synaptic strength (B; n = 5 cultures per group), indicating that granule cells utilize, at least in part, similar mechanisms to adjust their excitatory synaptic strength in a homeostatic manner following denervation and TTX-treatment.

A well established method to perturb neuronal activity and to induce homeostatic synaptic scaling is chronic blockade of sodium channels with TTX (e.g., [17]–[23]). We therefore combined denervation and TTX-treatment to test whether these two experimental conditions exploit, at least in part, similar mechanisms. A comparable approach to test for homeostatic synaptic scaling has been reported previously [24], [25]. If similar mechanisms are involved, we hypothesized that TTX should have no effect, or at least a smaller effect on mean mEPSC amplitude of denervated granule cells in comparison to non-denervated granule cells. If the underlying mechanisms are dissimilar, we expected that denervation and TTX should have an additive effect on granule cell mEPSC amplitudes. As shown in Figure 3, treatment of non-lesioned control cultures with 2 µM TTX for 2 d induced a significant increase in mEPSC amplitude, suggesting that dentate granule cells show robust synaptic up-scaling. The combination of denervation and TTX had no additive effect on the mean mEPSC amplitude (Figure 3; n = 5 cultures per group, 3–4 neurons recorded per culture).

### Layer-specific strengthening of excitatory synapses at 3–4 days post lesion

**Figure 4:**
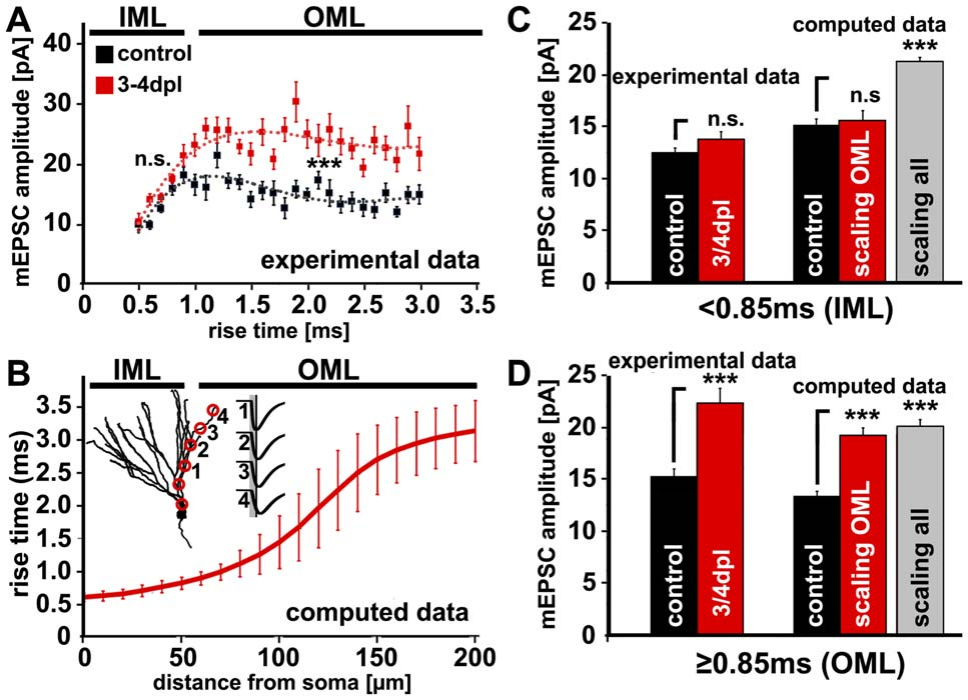
Rise time to distance from soma dependency indicated layer-specific changes in excitatory synaptic strength. (**A**) Sorting of mEPSC events by rise time revealed a significant difference between control and denervated dentate granule cells (at 3–4 dpl) for events with a rise time >0.85 ms. Shorter rise times did not show a significant difference. (**B**) Using compartmental modeling the rise time to distance from soma dependency was determined. Single mEPSCs were elicited at different locations along a dendrite and measured at the soma. Rise times of simulated mEPSCs increased with increasing distance of the activated input from the soma (n = 8 granule cells). A rise time of 0.85 ms corresponds to a distance of ∼50 µm from the soma, which in turn corresponds to the anatomical border between the inner molecular layer (IML) and the outer molecular layer (OML) of the dentate gyrus. (**C, D**) Experimental data and compartmental simulations of layer-specific scaling of excitatory synapses. Simulated mEPSCs were monitored at the soma of 8 model granule cells, in which synaptic background activity was elicited by random activation of dendritic synapses in the IML and OML. In agreement with mEPSC patch-clamp recordings, layer-specific scaling of OML synapses in model granule cells led to a significant increase of slow mEPSC amplitudes (rise times >0.85 ms). In contrast, scaling of model synapses in both layers (OML and IML) resulted in a significant increase of both slow and fast mEPSC amplitudes (rise times <0.85 ms). Thus, scaling of synapses in the denervated OML explained the experimental results illustrated in (A).

Since entorhinal denervation results in synapse loss in the OML [1], [2] we assessed whether the observed changes in synaptic strength were restricted to this zone. As the rise time of mEPSCs can be used to determine the distance of synapses from the soma of patched neurons [24], [26]–[29], we plotted the amplitude of mEPSCs recorded at 3–4 dpl against their rise time. This revealed that events with a slow rise time, which originate from distal dendrites, had significantly larger mean amplitudes following denervation compared to controls. In contrast, events with a fast rise time, which arise from proximal dendritic segments, did not exhibit increased mEPSC amplitudes. At a rise time of ∼0.85 ms the two groups separated (Figure 4A).

We next computed the rise time to distance from soma dependency using the compartmental model for mouse dentate granule cells published by Schmidt-Hieber and colleagues [30]. This model has been recently validated by the first dual somato-dendritic recordings of dentate granule cells by Krueppel et al. [31], who demonstrated that the properties of dentate granule cell dendrites can be well-described by the passive compartmental model of Schmidt-Hieber [30]. Using this model we found that synapses with a rise time of 0.85 ms are located at a distance of ∼50 µm from the granule cell soma (Figure 4B). Since a distance of 40–50 µm corresponds to the anatomical border between the IML and OML in mouse [32]–[36], these computations support the hypothesis that denervation leads to an increase in excitatory synaptic strength in the denervated OML.

The work by Krueppel and colleagues [31] also indicated the presence of low densities of voltage-dependent sodium channels and A-type potassium channels in the dendritic compartment of dentate granule cells. To verify that these active dendritic properties do not affect our computational results, we repeated the computations of the rise-time to distance from soma dependency using these additional parameters. These computations yielded similar result as illustrated in Figure 4B (see Figure S1).

Using the compartmental granule cell model of Schmidt-Hieber et al., [30], we subsequently simulated mEPSCs under two conditions: “global” strengthening of all synapses of a granule cell and “local” increase in synaptic strength only in the OML (Figure 4C, D; scaling all vs. scaling OML, n = 8 granule cells). Under both conditions every third synapse in the OML was silenced (switched off) to mimic spine loss following denervation (for details on spine density changes see [10]). This computational modeling approach revealed that a proportional strengthening of synapses in the OML by a factor of ∼1.5 (but not of all synapses) results in mEPSC amplitude changes that are similar to the ones we measured in our culture preparations after denervation.

### Local electrical stimulations confirm layer-specific strengthening of excitatory synapses after denervation

Since the validity of a computational modeling result depends on the quality of the model as well as on the specific condition for which the model was generated, we experimentally verified the predicted layer-specific strengthening of granule cell postsynapses. In particular, we were concerned that the granule cell model by Schmidt-Hieber et al. [30] is based on data obtained from acute hippocampal slices and not on data from organotypic entorhino-hippocampal slice cultures. Thus, we employed local electrical stimulations in Strontium (Sr^2+^)-containing bath solution [37], [38], which allowed us to test the strength of granule cell postsynapses in the denervated OML and the non-denervated IML. In this solution Ca^2+^ is partially replaced with Sr^2+^, which results in the asynchronous release of individual glutamate vesicles from presynaptic terminals in response to local electrical stimulation. The individual events that are recorded under these conditions correspond to quanta released and recorded under mEPSC-recording conditions. In contrast to conventional mEPSC recordings, which are based on a stochastic release of presynaptic vesicles, asynchronous EPSCs (aEPSCs) are induced by local electrical stimulation and can thus be attributed to the stimulated afferents [37], [38].

**Figure 5:**
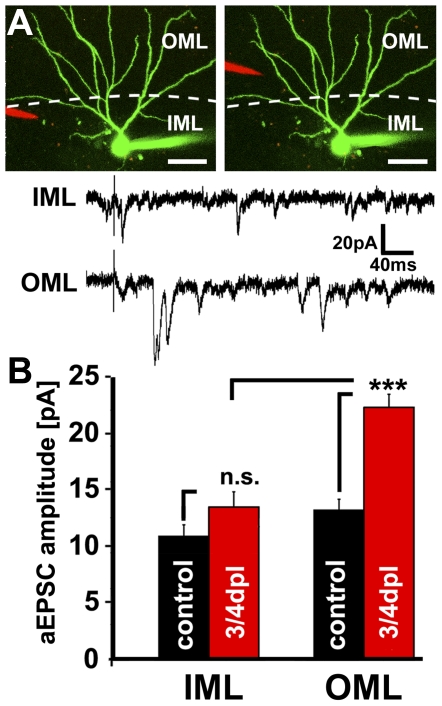
Electrically evoked asynchronous EPSCs confirmed layer-specific increase in excitatory synaptic strength. (**A**) Asynchronous EPSCs (aEPSC) were recorded from identified dentate granule cells in control and denervated cultures upon local electrical stimulation of the OML and IML (recordings performed in Sr^2+^-containing bath solution; red, pipette containing the stimulating electrode and Alexa568; green, patch-clamp electrode containing Alexa488). Scale bar: 40 µm. (**B**) An increase in mean aEPSC amplitude was observed in response to electrical stimulations of the OML but not in response to electrical stimulations of the IML of denervated dentate granule cells (n = 4 cultures each; 1–3 neurons per culture).

Accordingly, we stimulated the OML or the IML while recording asynchronous EPSCs (aEPSCs) [37], [38] from control or denervated dentate granule cells (at 3–4 dpl). Events within 400 ms after the stimulus were averaged for each neuron (Figure 5; n = 4 cultures per group; 1–3 neurons per culture; 20 stimulations at 0.1 Hz per layer). Indeed, high aEPSC amplitudes were only observed when the OML of denervated granule cells was stimulated. The mean amplitude of aEPSCs upon stimulation of the IML was not significantly different between control and denervated granule cells (Figure 5B). These data verified the modeling results and demonstrated that granule cells respond to entorhinal denervation with a layer-specific increase in excitatory synaptic strength at 3–4 dpl.

## Discussion

In the present study, we have addressed the hypothesis that neurons could respond to a denervation-induced loss of excitatory afferents with homeostatic up-scaling of their excitatory synaptic strength. We tested this hypothesis in organotypic entorhino-hippocampal slice culture preparations, in which we could reliably remove the excitatory entorhinal input to the distal dendrites of dentate granule cells, without injuring the cells themselves [9], [10]. Whole-cell patch-clamp recordings from identified granule cells revealed a denervation-induced increase in excitatory synaptic strength, which was first evident 6 h after denervation and lasted for at least 10 dpl. Of note, at 3–4 dpl changes in synaptic strength appeared to be restricted to the layer of denervation, suggesting that neurons which lose part of their input can adjust their excitatory synaptic strength locally. Taken together, our results provide the first experimental evidence for a denervation-induced homeostatic response of excitatory synaptic strength, which may compensate for the loss of afferent innervation.

### Changes in synaptic strength are inversely correlated with denervation-induced changes in spine density

Axonal denervation is a strong stimulus for a neuron to remodel its synapse population [1]. At the level of identified denervated granule cells several studies have shown that granule cells respond to entorhinal denervation with layer-specific changes in their spine density and a profound remodeling of their dendritic arbor [39]–[42]. We were recently able to confirm these findings using entorhino-hippocampal slice cultures of Thy1-GFP mice [9], [10]. In these culture preparations layer-specific spine density changes were seen: In the denervated OML spines were lost during the first week and spine density dropped to approximately 60–70% (at 4 dpl). During the second week after denervation spine density recovered.

At the functional level data on changes of identified granule cells after denervation are surprisingly scarce [43], [44]. Our study is the first, which has systematically studied changes in excitatory synaptic strength of denervated granule cells. Our results revealed a biphasic pattern in the change of mEPSC amplitudes following entorhinal deafferentation. The mean mEPSC amplitude increased significantly between 3 h and 6 h after denervation, reached a plateau between 1 dpl and 2 dpl and returned to the level of age-matched controls by 14 dpl (Figure 2D). This observation suggests an inverse interrelation between changes in synaptic strength and changes in spine density, and is thus in line with a compensatory, i.e., homeostatic response of granule cells following denervation.

The mEPSC frequencies did not follow the characteristic biphasic time-course of mEPSC amplitudes and spine density changes. An initial reduction in mEPSC frequencies was observed at 3 h following denervation, which subsequently recovered back to baseline and remained unchanged until 14 dpl (Figure 2E). Although a drop in mean mEPSC frequency was expected after the lesion, in line with a reduction of the number of presynaptic terminals, we were surprised by the speed of its recovery, which does not correspond to the structural recovery of synapses. This phenomenon cannot be explained at present, although it is likely that presynaptic homeostatic plasticity mechanisms (discussed in [4], [45]) could play a role.

### Changes in synaptic strength correlate with the loss and recovery of afferent innervation

Another form of structural plasticity, which has been described after entorhinal denervation is collateral sprouting of surviving axons [1], [2]. It has been proposed that axonal sprouting could compensate for the loss of entorhinal innervation and could contribute to the functional recovery of denervated dentate granule cells [1], [43]. Evidence for collateral sprouting of excitatory mossy cell axons from the inner molecular layer has been provided in organotypic slice cultures at 10 dpl [8]. This time point coincides with the observed reduction of excitatory synaptic strength back to control levels between 10 and 14 dpl in our experiments. It appears plausible that sprouting of excitatory afferents could result in an increased excitatory drive to denervated granule cells. This increase in excitatory drive could, in turn, lead to a homeostatic reduction in synaptic strength. Thus, changes in synaptic strength may mirror denervation and reinnervation, i.e., the loss of afferent input correlates with an increase in excitatory synaptic strength and the recovery of afferent input correlates with the decrease in synaptic strength. Together these observations suggest that changes in the synaptic strength of denervated granule cells are closely linked to granule cell innervation.

### Denervation-induced increase in synaptic strength is comparable to TTX-induced homeostatic synaptic scaling

Homeostatic synaptic scaling is a plasticity mechanism which stabilizes the activity of a neuron in the face of perturbations, such as alterations in afferent input [4], [5]. Although molecularly distinct from classical Hebbian forms of plasticity, homeostatic synaptic scaling is known to depend on changes in AMPA receptor synthesis and accumulation at excitatory postsynapses (e.g., [17], [21], [22], [46], [47]). A classical experimental approach to induce homeostatic synaptic up-scaling of excitatory synapses is a prolonged blockade of neural activity with TTX [14]. This leads to a compensatory strengthening of excitatory synapses which preserves the relative weight between individual synapses and aims at keeping the firing rate of a neuron within a dynamic range [14].

In the present study, we wondered whether the observed denervation-induced increase in synaptic strength could resemble homeostatic synaptic scaling as seen after TTX-treatment. This appeared to be a possibility, since both, TTX-treatment as well as entorhinal denervation reduces the excitatory drive to dentate granule cells. Accordingly, we combined denervation and TTX-treatment, in an approach similar to the one used by other groups [24], [25]. We predicted that the effect of the two treatments should not be additive, if similar mechanisms are involved. Our experiments indeed showed that the two treatments are not additive at 4 dpl (Figure 3) and thus demonstrated that entorhinal denervation induces an increase in excitatory synaptic strength that appears to be in line with a homeostatic synaptic scaling response observed in other experimental conditions.

### Entorhinal denervation induces layer-specific homeostatic synaptic up-scaling of granule excitatory postsynapses at 3–4 days post lesion

A major strength of the entorhinal denervation model is the laminar termination of afferents to the dentate gyrus [48]. Entorhinal fibers terminate in the OML, while associational fibers terminate in the IML of the dentate gyrus. Since entorhinal denervation removes entorhinal fibers from the OML while leaving associational fibers to the IML intact, the effects of a partial and layer-specific denervation of dentate granule cells can be studied [1], [2]. This anatomical organization made it possible to address the question, whether partial denervation after entorhinal lesion elicits global changes in the excitatory synaptic strength of granule cells or local changes in the synaptic strength of surviving synapses within the layer of denervation. In the former case we predicted that entorhinal denervation should increase the strength of proximal as well as distal synapses, in the latter case we predicted that entorhinal denervation should increase the strength of distal synapses only.

To address this question experimentally, we employed mEPSC rise time analysis, computational modeling and layer-specific electrical stimulations in Sr^2+^-containing bath solution (Figures 4 and 5). Together, these approaches provided evidence for a lamina-specific increase in excitatory synaptic strength 3–4 days following entorhinal denervation. This observation is in line with a set of publications, which have shown that neurons can scale their synapses locally (e.g., [19], [23], [47], [49]–[51]; for review see [52]). Thus, following entorhinal denervation granule cells appear to increase the strength of their surviving distal synapses, which are located in the denervated OML, while maintaining the strength of their proximal synapses, which are located in the non-denervated IML. It remains to be shown, however, whether this ability of granule cells to scale their excitatory postsynapses in a layer-specific fashion is a property of the target cell or afferent-specific, as has been shown by the group of Tsien [19], [23] for the CA3 region of the hippocampus.

### What are the molecular mechanisms which control denervation-induced homeostatic synaptic scaling?

The signals that mediate layer-specific responses of dentate granule cells to denervation are not understood. Evidence has been provided for neuron-glia interactions, where glial cells regulate critical steps of the reorganisation process [53]–[55]. Following entorhinal denervation glial cells delineate the denervated zone [56]–[61] and are thus in the correct spatial position to provide lamina-specific regulatory cues to denervated granule cells. Since earlier reports have shown that astrocyte-derived tumor necrosis factor alpha (TNFα) plays a role in synaptic scaling by regulating the accumulation of AMPA receptors at the cell surface [25], [62]–[65], it appears to be plausible that TNFα could also be involved in the denervation-induced homeostatic scaling response. The time-course of mEPSC changes following entorhinal denervation reported in the present study will be useful in future work focussing on the role of TNFα and other candidate regulatory molecules implicated in homeostatic up- (week 1 following denervation) or down-scaling (week 2 following denervation) of excitatory synaptic strength.

### Homeostatic synaptic scaling and its role in diseases associated with neuron or synapse loss

Homeostatic synaptic scaling has recently been discussed in the context of neurological diseases. In particular, it has been suggested that synaptic scaling may be a homeostatic mechanism that could come into play following the loss of synapses or neurons [66]–[68]. Our data support this hypothesis as we observed a compensatory increase in excitatory synaptic strength following denervation. Since denervation of brain regions connected with lesion sites occurs in a large number of neurological diseases, it appears highly plausible that a broad range of neurological diseases will be accompanied by compensatory, i.e., homeostatic, responses of denervated neurons.

The biological consequences of lesion-induced homeostatic synaptic scaling for the course of a neurological disease are not yet understood. On the one hand, it has been proposed that homeostatic synaptic scaling could counteract the disease-induced loss of afferent input to neurons by keeping the activity of the affected neuron within a physiological range [66]. Such a mechanism could be beneficial and could retard memory loss in Alzheimer's disease. On the other hand it has been also discussed that epilepsy could be a consequence of homeostatic plasticity [69]–[71]. The entorhinal cortex lesion model (in vivo and in vitro) may be an ideal tool to address these important questions in future work.

## Materials and Methods

### Ethics statement

Animal care and experimental procedures were performed in agreement with the German law on the use of laboratory animals (animal welfare act; TierSchG; §4 Abs. 3) and approved by the animal welfare officer of Goethe-University, Faculty of medicine (reference number BB01/10/2011).

### Preparation of slice cultures

Entorhino-hippocampal slice cultures were prepared at postnatal day 4–5 from Thy1-GFP mice with a C57BL/6J background of either sex as previously described [7], [10], [72]. Cultivation medium contained 50% MEM, 25% basal medium eagle, 25% heat-inactivated normal horse serum, 25 mM HEPES buffer solution, 0.15% bicarbonate, 0.65% glucose, 0.1 mg/ml streptomycin, 100 U/ml penicillin, and 2 mM glutamax. pH was adjusted to 7.3 and medium was replaced every second day. All slice cultures were allowed to mature for 18–20 days in humidified atmosphere with 5% CO_2_ at 35°C.

### Entorhinal cortex lesion

Slice cultures (18–20 div) were completely transected from the rhinal fissure to the hippocampal fissure using a sterile scalpel blade. To ensure complete and reproducible separation of the entorhinal cortex from the hippocampus, the entorhinal cortex was removed (Figure 1B) in every denervation experiment.

### Entorhinal fiber tracing and nuclear staining

A small cristal of Mini-Rubi [10], [73] was inserted into the entorhinal cortex of 3 weeks old slice cultures using a patch pipette. Cultures were returned to the incubator and fixed 3 d later in a solution of 4% (w/v) paraformaldehyde (PFA) in phosphate buffered saline (PBS, 0.1 M, pH 7.4) and 4% (w/v) sucrose for 1 h, followed by washing in PBS and counterstained with TO-PRO nuclear stain (1∶5000 in PBS for 10 min). Cultures were washed again, transferred onto glass slides and mounted for visualization with anti-fading mounting medium. Confocal images were acquired using a Nikon Eclipse C1si laser-scanning microscope with a 10× (numerical aperture, NA 0.30, Nikon) and a 60× oil-immersion (NA 1.3, Nikon) objective lens, respectively. Detector gain and amplifier were initially set to obtain pixel densities within a linear range. All images were sampled with ideal Nyquist rate.

### Whole-cell patch-clamp recordings

Whole-cell voltage-recordings from dentate granule cells were carried out at 35°C as previously described [74]. The bath solution contained 126 mM NaCl, 2.5 mM KCl, 26 mM NaHCO_3_, 1,25 mM NaH_2_PO_4_, 2 mM CaCl_2_, 2 mM MgCl_2_, and 10 mM glucose. Patch pipettes contained 126 mM K-gluconate, 4 mM KCl, 4 mM ATP-Mg, 0.3 mM GTP-Na_2_, 10 mM PO-Creatine, 10 mM HEPES and 0.3% Biocytin (pH = 7.25 with KOH, 290 mOsm with sucrose) having a resistance of 6–10 MOhm. Dentate granule cells were recorded at −70 mV in the presence of 10 µM D-AP5, 10 µM SR-95531 and 0.5 µM TTX. Neurons were post hoc identified using Alexa568-conjugated streptavidin (Invitrogen, 1∶500 in PBS, 1% NGS, 0.2% Triton X-100); on the basis of this analysis (identification of basal dendrites and/or axonal projections within the dentate gyrus) 12 recorded neurons were excluded from analysis in the present study. In all experiments series resistance was monitored in 2–3 min intervals, and recordings were discarded if the series resistance reached 30 MΩ.

#### Time course of synaptic scaling after denervation

In this experiment n = 4–11 cultures per group with ≥3 neurons per culture (165 neurons total) were recorded. Mean values of recorded neurons were averaged per culture. Age-matched non-lesioned control cultures prepared from the same animal or littermate animals served as controls. Control cultures were recorded alternating with the recordings of denervated cultures at 3 h, 6 h, 12 h and 24 h (control cultures of these time points were pooled; data shown as 0/1 d in Figure 2D, E), as well as for 3–4 dpl, 10 dpl and 14 dpl. Recordings at 48 h and 7 dpl were not accompanied by age-matched non-lesioned control data. Statistical comparison between controls (0–1 d with 3–4 d; 3–4 d with 10 d or 14 d) revealed no significant difference in the mEPSC properties of control neurons at these ages. ≥8 independent litters were used in this set of experiments. The resting membrane potential was not significantly different between the groups (e.g., control, −76.3±1.8 mV; 3 h, −74.7±0.9 mV; 12 h, −75.3±1.3 mV; 24 h, −74.7±1.6 mV; 3–4 dpl, −73.4±4.6 mV; 7 dpl, −78.1±2.0 mV; 14 d −75.5±1.2 mV).

#### TTX treatment of denervated cultures

In these experiments n = 5 cultures were recorded per group, accompanied by age-matched non-lesioned control cultures (see above). TTX (2 µM) was added to the incubation medium at 2 dpl for 2 days.

#### Local electrical stimulations in Sr^2+^-containing bath solution

Local electrical stimulations were carried out in Strontium (Sr^2+^)-containing bath solution (4 mM SrCl_2_, 1 mM CaCl_2_, 2 mM MgCl_2_, 126 mM NaCl, 2.5 mM KCl, 10 mM HEPES and 10 mM glucose; pH = 7.3 with NaOH), which leads to an evoked asynchronous release of presynaptic glutamate vesicles. Previous work [37], [38] has shown that these locally evoked events compare to mEPSCs, and can be used to assess the postsynaptic strength of synapses on which the electrically stimulated axons terminate. Glass pipettes (6–10 MOhm; filled with bath solution and 10 µM Alexa568) holding the stimulation electrode were positioned in the OML or IML (>20 µm apart from closest dendritic branch) and current pulses (4 µA; 500 µs; 20 at 0.1 Hz each layer) were generated by a stimulus generator STG1002 (Multichannel Systems, Reutlingen, Germany) while recording evoked asynchronous EPSCs from individual granule cells (age-matched controls vs. 3–4 dpl) in whole-cell voltage-mode (events within a 400 ms following stimulation were analyzed). The position of the stimulation pipette was documented in confocal image stacks. Recorded neurons were filled with Alexa488 (10 µM) in these experiments (n = 4 cultures per group; 1–3 neurons recorded per culture).

#### Entorhinal cortex stimulation

A bipolar stimulation electrode (NE-200, 0.5 mm tip separation, Rhodes Medical Instruments, Wood hills, CA) was positioned in layer 2–3 of the entorhinal cortex and current pulses (50 µA; 1 ms) were generated by a stimulus generator STG1002 (Multichannel Systems, Reutlingen, Germany) while recording from individual granule cells in whole-cell mode (n = 5 cultures recorded at 18–20 div and at 34 div).

### Compartmental modeling

Compartmental simulations were performed using 8 morphologically realistic mouse dentate granule cells [30] which were downloaded from http://senselab.med.yale.edu/ModelDB/ShowModel.asp?model=95960. Model granule cells were implemented in the simulation environment NEURON ([75]; www.neuron.yale.edu). Realistic passive properties were taken from Schmidt-Hieber et al. [30]. Excitatory (AMPA receptor-mediated) synaptic conductance changes were simulated using the sum of two exponential functions [30]: rise time 0.2 ms; decay time 2.5 ms; peak conductance 0.5 nS; reversal potential 0 mV.

### Rise time to distance from soma dependency

To determine the distribution of simulated mEPSC rise times with increasing distance from the granule cell layer, identical single synaptic input was activated at different locations along a path between the soma and a distal end of the dendrite, and corresponding mEPSCs were detected at the soma. Simulated cells were voltage clamped at −70 mV and an explicit series resistance of 2 MΩ was included. In some experiments (see Figure S1) the properties of these mouse model granule cells were modified according to Krueppel et al. [31], by inserting a low concentration of voltage-dependent sodium channels (1 mS/cm^2^) and A-type potassium channels (10 mS/cm^2^).

### Simulations of global and local scaling

To simulate the effects of scaling of dendritic synapses on mEPSCs, we monitored voltage-clamped somatic currents in 8 model granule cells [30] in which synaptic background activity was arising from the random (Poisson) low frequency activation (0.017 Hz) of dendritic AMPA synapses in the inner and outer molecular layer (IML/OML). In these simulations, AMPA synapses were placed on all spines of granule cell OML and IML dendrites. Spine numbers and densities for spine-bearing compartments of 8 granule cells (average spine density: 2.39±0.06/µm) were taken from Schmidt-Hieber et al. [30]. Spines were implicitly implemented into the model by scaling the specific membrane resistance (Rm) and the specific membrane capacitance (Cm) of dendritic compartments [30]. In each cell, all synapses proximal to the IML/OML border (specified as somatic length/2+44 µm) were defined as IML synapses. All synaptic inputs located between the IML and the ends of distal dendrites were defined as OML synapses. We used the same kinetic parameters for dendritic OML/IML AMPA synapses as in single mEPSC simulations: rise time 0.2 ms; decay time 2.5 ms. In simulations of the synaptic scaling, following synaptic parameters were used: reversal potential 0 mV; control mean peak conductance for synapses in the IML and the OML 0.25 nS (100%) and 0.5 nS (100%), respectively; scaled mean peak conductance for synapses in the IML and the OML: 0.375 nS (150%) and 0.75 nS (150%), respectively; holding voltage −70 mV; series resistance 2 MΩ. To add variability to synaptic events, the conductance values were sampled randomly from Gaussian distributions with respective peak conductance means (see above) and a variance of 2.5×10^−6^ nS. In “scaling OML” simulations, proportional (150%) scaling of OML synapses was implemented and every third OML synapse was silenced (switched off) to mimic the spine loss following ECL (spine density is reduced down to approximate 60–70% at 4 dpl; [10]). In “scaling all” simulations the same proportion of synapses was switched off in OML, but proportional (150%) scaling of OML as well as IML synapses was used. Note that in control (unscaled) conditions, larger conductance was used for OML synapses as compared to IML synapses as patch-clamp mEPSC data suggested larger synaptic strength of distal dendritic inputs in comparison to proximal inputs (amplitudes of slow mEPSCs with rise times >0.85 ms were larger than amplitudes of fast mEPSCs with rise times <0.85 ms). Analysis of amplitudes and rise times of simulated mEPSCs was performed using an automatic detection software MiniAnalysis (Synaptosoft, USA).

### Quantification and statistics

Electrophysiological data were analyzed using pClamp 10.2 (Axon Instruments, USA) and MiniAnalysis (Synaptosoft, USA) software. All events were visually inspected and detected by an independent investigator blind to experimental condition. 300–400 events were analyzed per recorded neuron. Evoked asynchronous mEPSC were analyzed within 400 ms after each stimulus (20 at 0.1 Hz in each layer). Statistical comparisons were made using Wilcoxon-Mann-Whitney test followed by Bonferroni's correction where appropriate. P-values of less than 0.05 were considered a significant difference. All values are expressed as mean ± SEM.

### Digital Illustrations

Confocal image stacks were exported as 2D-projections and stored as TIFF files. Figures were prepared using Photoshop CS2 graphics software (Adobe, San Jose, CA, USA). Image brightness and contrast were adjusted.

## Supporting Information

Figure S1 **Comparison of rise time to distance from soma dependencies using the passive model by Schmidt-Hieber et al. (2007) and the active model by Krueppel et al. (2011).** Krueppel et al., (2011, [31]) assessed dendritic properties of rat dentate granule cells using dual somato-dendritic patch-clamp recordings. Although their results were in agreement with the passive granule cell model of Schmidt-Hieber et al. (2007, [30]), these authors also reported a low concentration of A-type potassium currents (10 mS/cm2) and transient sodium currents (1 mS/cm2) in the dendritic compartment of granule cells. To exclude the possibility that these active channels could influence the results of our computations, we repeated the rise time to distance from soma dependency simulations using the dendritic properties determined by Krueppel et al. (red; granule cell 7 from Schmidt-Hieber et al., [30]). This yielded the same results as seen in the passive model of Schmidt-Hieber et al. (black; granule cell 7). The rise time to distance from soma dependency was not affected by the systematic variation of synaptic strength from 0.25 nS to 1 nS (data not shown).
(TIF)
