# Supplementary figures and images for: Entorhinal Denervation Induces Homeostatic Synaptic Scaling of Excitatory Postsynapses of Dentate Granule Cells in Mouse Organotypic Slice Cultures
Source: PLoS One. 2012 Mar 5;7(3):e32883. doi: 10.1371/journal.pone.0032883 (PMC3293910; doi:10.1371/journal.pone.0032883)

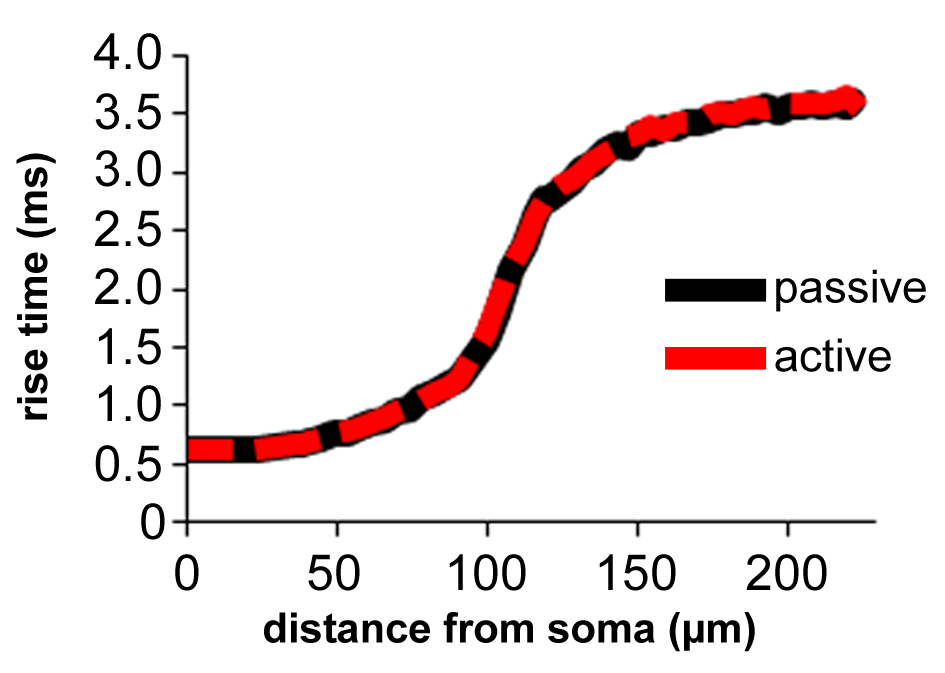

Supplement: Figure S1 — Comparison of rise time to distance from soma dependencies using the passive model by Schmidt-Hieber et al. (2007) and the active model by Krueppel et al. (2011). Krueppel et al., (2011, [31]) assessed dendritic properties of rat dentate granule cells using dual somato-dendritic patch-clamp recordings. Although their results were in agreement with the passive granule cell model of Schmidt-Hieber et al. (2007, [30]), these authors also reported a low concentration of A-type potassium currents (10 mS/cm2) and transient sodium currents (1 mS/cm2) in the dendritic compartment of granule cells. To exclude the possibility that these active channels could influence the results of our computations, we repeated the rise time to distance from soma dependency simulations using the dendritic properties determined by Krueppel et al. (red; granule cell 7 from Schmidt-Hieber et al., [30]). This yielded the same results as seen in the passive model of Schmidt-Hieber et al. (black; granule cell 7). The rise time to distance from soma dependency was not affected by the systematic variation of synaptic strength from 0.25 nS to 1 nS (data not shown). (TIF) [file pone.0032883.s001.tif]
